# Crosstalk between Metabolic Alterations and Altered Redox Balance in PTC-Derived Cell Lines

**DOI:** 10.3390/metabo9020023

**Published:** 2019-02-01

**Authors:** Laura Tronci, Paola Caria, Daniela Virginia Frau, Sonia Liggi, Cristina Piras, Federica Murgia, Maria Laura Santoru, Monica Pibiri, Monica Deiana, Julian Leether Griffin, Roberta Vanni, Luigi Atzori

**Affiliations:** 1 Department of Biomedical Sciences, University of Cagliari, 09124 Cagliari, Italy; paola.caria@unica.it (P.C.); dvfrau@unica.it (D.V.F.); sl584@cam.ac.uk (S.L.); cristina.piras@unica.it (C.P.); federica.murgia@unica.it (F.M.); marialaurasantoru@gmail.com (M.L.S.); mpibiri@unica.it (M.P.); mdeiana@unica.it (M.D.); vanni@unica.it (R.V.); latzori@unica.it (L.A.); 2 Department of Biochemistry and Cambridge Systems Biology Centre, University of Cambridge, Cambridge CB2 1GA, UK; jlg40@cam.ac.uk

**Keywords:** papillary thyroid carcinoma, metabolomics, metabolic profile, oxidative stress, cancer cell metabolism

## Abstract

**Background**: Thyroid cancer is the most common endocrine malignancy, with papillary thyroid carcinoma (PTC) being the most common (85–90%) among all the different types of thyroid carcinomas. Cancer cells show metabolic alterations and, due to their rapid proliferation, an accumulation of reactive oxygen species, playing a fundamental role in cancer development and progression. Currently, the crosstalk among thyrocytes metabolism, redox balance and oncogenic mutations remain poorly characterized. The aim of this study was to investigate the interplay among metabolic alterations, redox homeostasis and oncogenic mutations in PTC-derived cells. **Methods**: Metabolic and redox profile, glutamate-cysteine ligase, glutaminase-1 and metabolic transporters were evaluated in PTC-derived cell lines with distinguished genetic background (TPC-1, K1 and B-CPAP), as well as in an immortalized thyroid cell line (Nthy-ori3-1) selected as control. **Results**: PTC-derived cells, particularly B-CPAP cells, harboring BRAF, TP53 and human telomerase reverse transcriptase (hTERT) mutation, displayed an increase of metabolites and transporters involved in energetic pathways. Furthermore, all PTC-derived cells showed altered redox homeostasis, as reported by the decreased antioxidant ratios, as well as the increased levels of intracellular oxidant species. **Conclusion**: Our findings confirmed the pivotal role of the metabolism and redox state regulation in the PTC biology. Particularly, the most perturbed metabolic phenotypes were found in B-CPAP cells, which are characterized by the most aggressive genetic background.

## 1. Introduction

Metabolic reprogramming in tumor has been reported as one of the “hallmarks of cancer”, providing proliferating cancer cells with the bioenergetic and biosynthetic metabolites and biosynthetic advantages needed for neoplastic transformation [1]. This adaptation results in dysregulation of several pathways, including glycolysis, glutaminolysis, citric acid cycle, pentose phosphate shunt, and lipid metabolism [2]. A close relationship between these alterations in cancer metabolism and both oncogenes and tumor suppressors signaling is indicated by a number of evidences [3]. Additionally, malignant transformation in cancer cells has been largely associated with increased reactive oxygen species (ROS) levels [4,5], which in the tumor microenvironment are responsible for the activation of signaling pathways (PIP3K, MAPK/ERK, HIF, and NF-Kb signaling pathways) necessary for tumorigenesis and cell survival [6]. Oncogenic mutations and metabolic alterations allow cancer cells to counteract the oxidative stress resulting from the accumulation of ROS by upregulating the antioxidant system mainly due the activity of transcriptional targets such as the nuclear factor (erythroid-derived 2)-like 2 (NRF2) [6] and overexpressing enzymes involved in the biosynthesis of antioxidant species, such as reduced glutathione (GSH) and cysteine [7].

The resulting delicate balance between intracellular ROS and antioxidant needs is closely dependent on various metabolic pathways, generating a crosstalk between metabolism and redox homeostasis [8].

Amongst the family of endocrine cancers, thyroid carcinomas is the most frequent manifestation. The majority of thyroid tumors are differentiated forms, with 85–90% of them represented by papillary thyroid carcinoma (PTC) [9]. Recurrent somatic genetic alterations in known oncogenes have been identified in PTC, with *BRAF*, *RAS*, and *RET*/*PTC* rearrangements occurring in 29–83%, 10–20%, and ~20% of PTC, respectively [9,10,11]. Moreover, *hTERT*) promoter mutations, conferring increased promoter activity of the *TERT* gene, have been observed in approximately 11% of PTC with aggressive behavior [12], and together with other genetic alterations, such as *TP53* mutations, are found to be associated with aggressive forms of PTC [13].

*BRAF^V600E^* mutation and *RET* rearrangements lead to constitutive activation of MAPK signaling pathway, which mostly regulates cell growth, differentiation, and survival [10]. Although genomics, transcriptomics and proteomics studies have contributed to a better understanding of PTC, they do not completely characterize the cancer phenotype closer to the cancer metabolome and redox balance [14]. To the best of our knowledge, there is no evidence yet about a possible connection between altered metabolism, redox homeostasis and the different genetic backgrounds in PTC. In this work, we investigate the metabolic changes and the redox status of three PTC-derived cell lines (TPC-1, K1, and B-CPAP), carrying a different genetic background. An immortalized normal thyrocytes cell line Nthy-ori3-1, that is negative for the aforementioned PTC genetic mutations, was used for comparison (Table 1).

## 2. Results

### 2.1. Metabolomic Profiles of PTC-Derived Cell Lines

**Figure 1:**
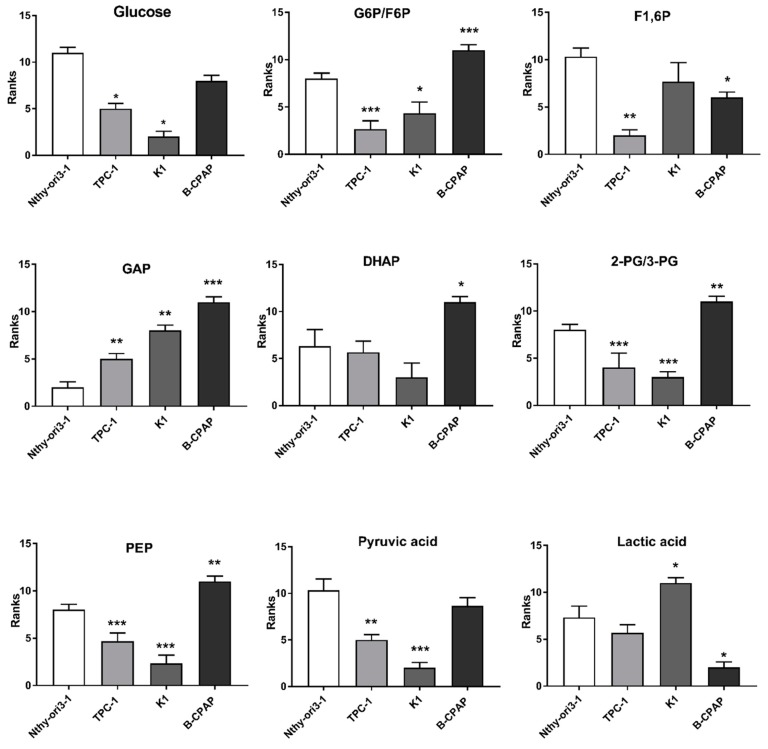
**Glycolytic Pathway in PTC-derived cells**. Metabolic alterations in glycolysis measured using ultra-high performance liquid chromatography–tandem mass spectrometry (UHPLC-MS/MS). Bar graphs indicate the relative concentration (peak areas) of the metabolites expresses as ranks. All experiments were performed three times independently, each time in triplicate to confirm the results. Statistical analyses were performed by Student *t*-test. Results were considered significant when * *p* < 0.05, ** *p* < 0.01, *** *p* < 0.001.

**Figure 2:**
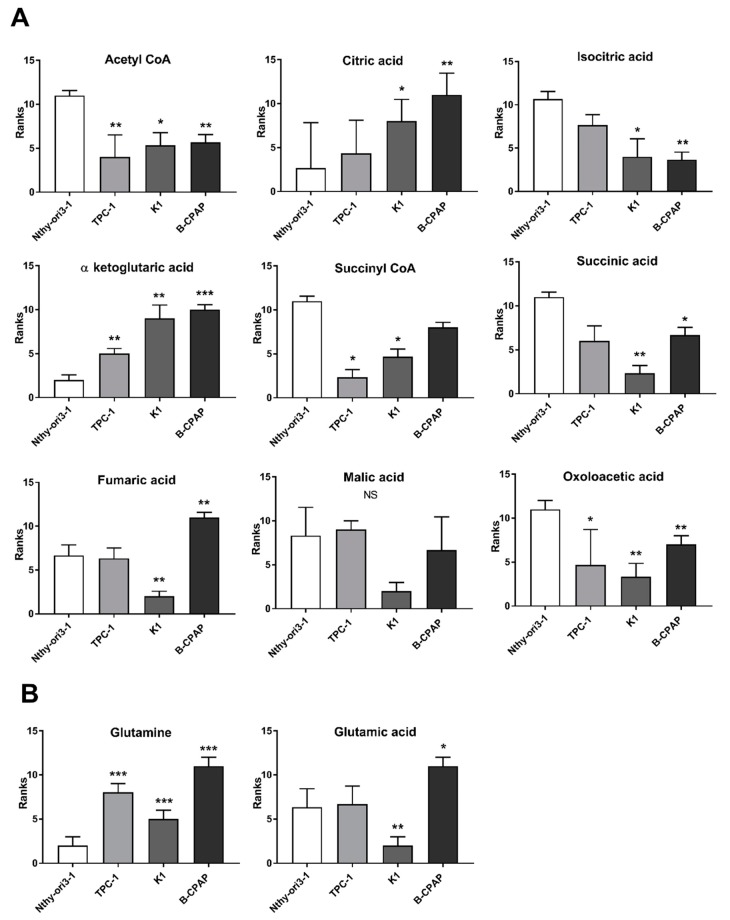
**Tricarboxylic acid cycle (TCA) and glutaminolysis pathways in PTC-derived cells**. Metabolic alterations in TCA cycle (**A**) and glutaminolysis (**B**) measured using UHPLC-MS/MS. Bar graphs indicate the relative concentration of the metabolites. All experiments were performed three times independently, each time in triplicate to confirm the results. Statistical analyses were performed by Student *t*-test. Results were considered significant when * *p* < 0.05, ** *p* < 0.01, *** *p* < 0.001.

Metabolites, involved in glycolysis, tricarboxylic acid (TCA) cycle, and glutaminolysis, play a crucial role in the metabolic adaptation of cancer cells. Data obtained from the metabolomic targeted analysis were compared to the “control” cells, to evaluate significantly changes in metabolites. *p* values, obtained from Student *t*-test, were corrected by using Holm–Bonferroni sequential correction method (Table S1). The data obtained from of PTC-derived cells were compared to the Nthy-ori3-1cells, used as control, to evaluate significant changes in metabolites. The results revealed an increase of metabolites associated with glycolysis [Glucose-6-phosphate/fructose-6-phosphate (G6P/F6P), dihydroxyacetone phosphate (DHAP), glyceraldheide-3-phosphate (GAP), 2/3-phosphoglycerate (2/3PG), and phosphoenolpyruvate (PEP)] and a decrease of fructose-1,6-phosphate (F1, 6P) and lactate in B-CPAP compared to both control and other PTC-derived cells (Figure 1). Glucose and pyruvate were significantly decreased only in TPC-1 and K1 compared to control cells. Similarly, fumaric acid was increased only in the B-CPAP cell line, while the other members of the TCA cycle, namely citric acid and α-ketoglutarate, were significantly increased in B-CPAP, K1, and TPC-1 cells compared to controls. Conversely, acetyl CoA, isocitric acid, succinyl-CoA, succinic acid, and oxaloacetate levels were significantly decreased in all cancer cells (Figure 2A). Regarding the glutaminolysis pathway, glutamine was increased in all cancer cells, while glutamate was significantly increased only in B-CPAP and significant decreased in K1 compared to control cells (Figure 2B).

### 2.2. Expression of GLUT1 and MCT4 Transporters and Glucose Uptake Results 

**Figure 3:**
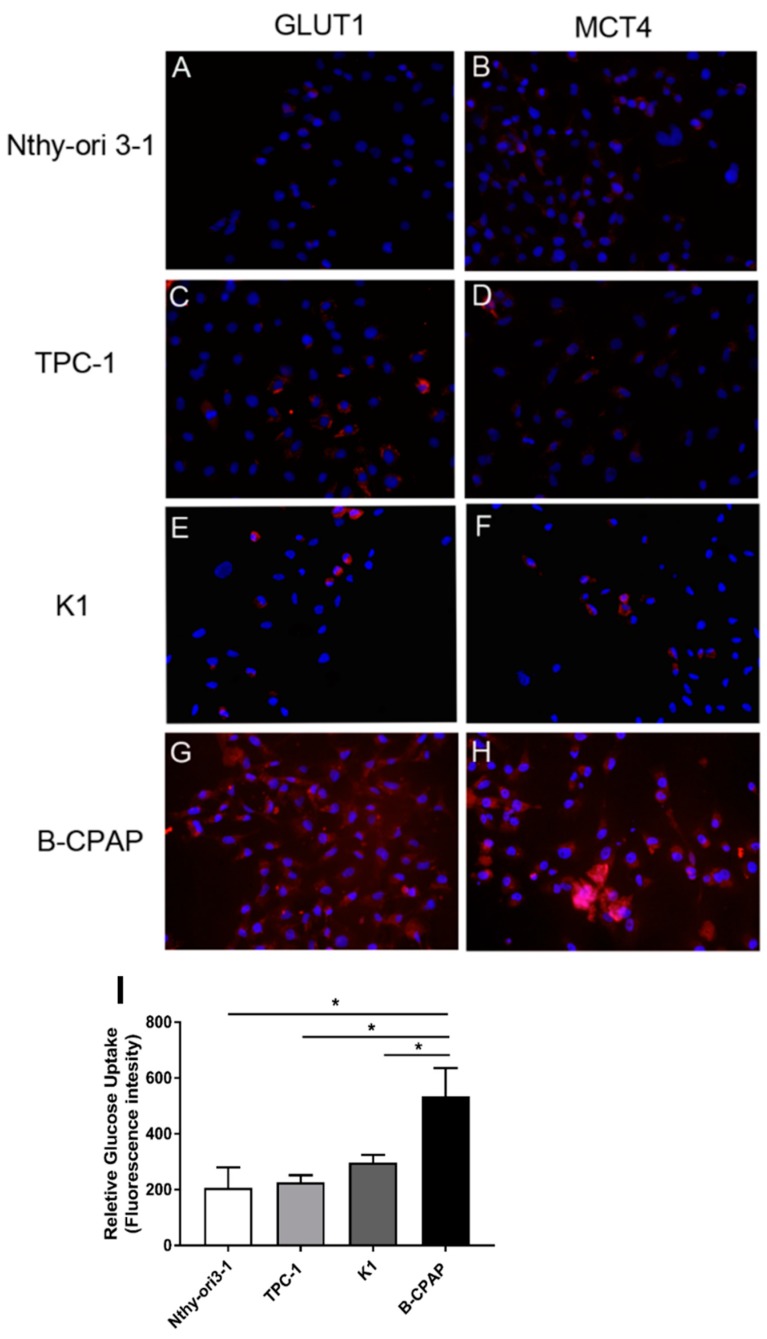
**Expression of GLUT-1, MCT-4 and glucose uptake in PTC-derived cells**. Immunofluorescence pattern for GLUT1 and MCT4 in Nthy-ori3-1 (**A**,**B**), TPC-1 (**C**,**D**), K1 (**E**,**F**) and B-CPAP (**G**,**H**). Nuclei were stained with DAPI (blue). Quantification of the relative glucose uptake was performed through the fluorescent glucose analog 2-NBDG in all cell lines (**I**). Data are expressed as media ± SD. All experiments were performed three times independently, each time in triplicate to confirm the results. Statistical analyses were performed by Student *t*-test. Results were considered significant when * *p* < 0.05, ** *p* < 0.01, *** *p* < 0.001.

To better characterize changes in the energetic mechanisms of PTC-derived cells, immunofluorescence analysis of the two transporters for glucose (GLUT1) and lactic acid (MCT4) was performed along with glucose uptake measurement using the fluorescent glucose analog 2-NBDG. These analyses showed Nthy-ori3-1 and TPC-1 cells were barely positive for both carriers expression (Figure 3A,B) while K1 and, mostly, B-CPAP cells were positive for GLUT1 and MCT4 (Figure 3E–H). Similarly, only B-CPAP cells showed a significantly increased glucose uptake (Figure 3I).

### 2.3. Redox Alterations in PTC Cells 

**Figure 4:**
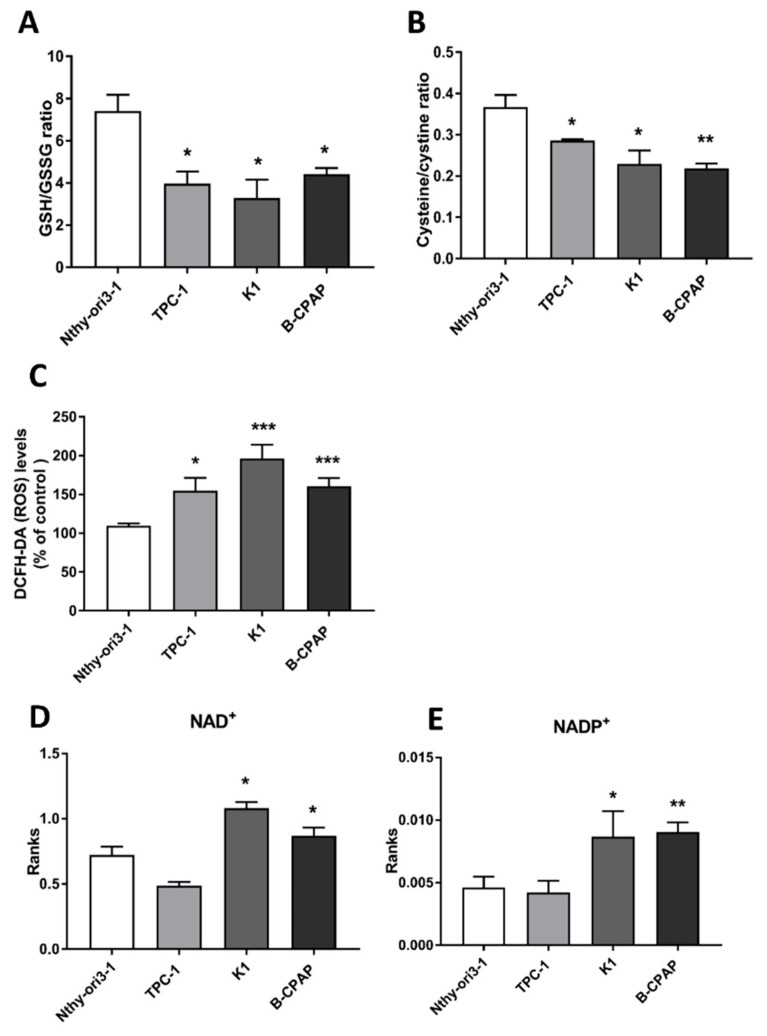
**Redox control in human thyroid cell lines**. (**A**) GSH/GSSG ratio in human thyroid cell lines. (**B**) Levels Cysteine/Cystine ratio in human thyroid cell lines. (**C**) Oxidant level in human thyroid cancer cell lines, expressed as % of control (human thyroid cell line, Nthy-ori3-1) (**D**) Intracellular NAD^+^ levels. (**E**) Intracellular NADP^+^ levels detected by UHPLC-MS/MS targeted analysis. Data are presented as means ± SD). All experiments were performed three times independently, each time in triplicate to confirm the results. Statistical analysis were performed by Student *t*-Test. Results were considered significant when * *p* < 0.05, ** *p* < 0.01, *** *p* < 0.001.

Redox balance is a crucial feature in the tumor development and maintenance. In order to evaluate any possible difference in its regulation and maintenance in our cell lines, we measured antioxidants species, ROS levels, and electron carriers. More specifically, intracellular aminothyols, expressed as ratio of reduced/oxidized glutathione and cysteine/cystine, were detected in PTC-derived cells through high pressure liquid chromatography (HPLC) coupled with an electrochemical detector (ECD). Levels of GSH/GSSG ratio were found to be significantly decreased in B-CPAP, K1 and TPC-1 cancer cells compared to control cells (Figure 4A). The same trend was observed for the cysteine/cystine ratio, which was significantly decreased in all cancer cells lines when compared to control (Figure 4B). Intracellular Oxidant species were measured by using 2’,7’-dichlorofluorescein diacetate (H^2^-DCF-DA) probe, which is the most widely used method to give a general measurement of oxidant production in the cells, although it does not provide a specific information about the type of oxidant. Oxidant levels were significantly increased in cancer cells compared to control cells (Figure 4C). Furthermore, NAD^+^ and NADP^+^ intracellular levels, measured by ultra-high performance liquid chromatography–tandem mass spectrometry (UHPLC-MS/MS), were significant increased in B-CPAP and K1 cancer cell lines compared to control (Figure 4D,E).

### 2.4. Expression of Glutamate Cysteine Ligase and Glutaminase 1

**Figure 5:**
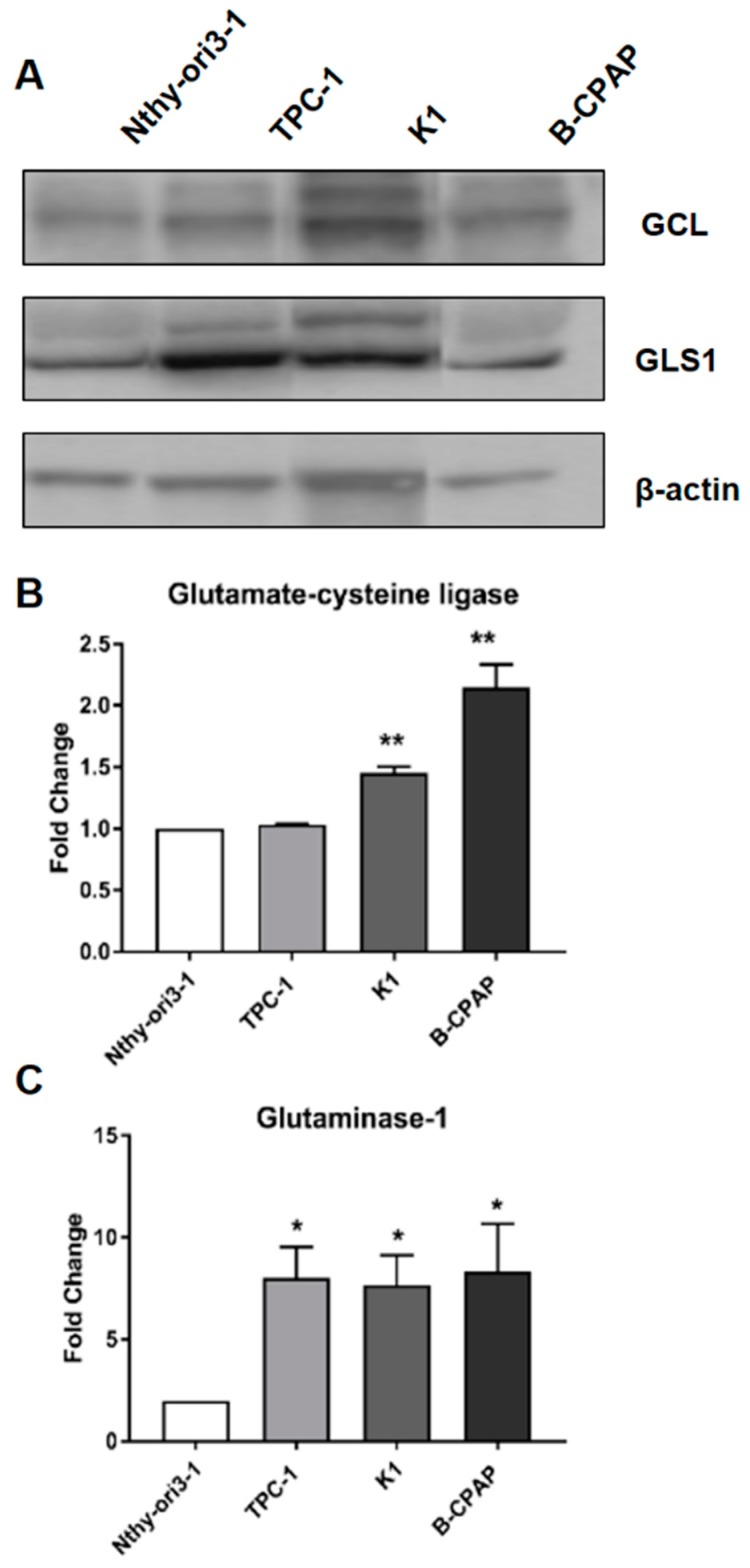
**Expression of glutamate-cysteine ligase and glutaminase 1**. (**A**) Western blot of both glutamate-cysteine ligase (GCL) and GLS1 with the reference gene β-actin. (**B**) Expression of glutamate-cysteine ligase in PTC-derived and control cells. (**C**) Expression of glutaminase-1 in PTC-derived and control cells. Data are expressed as fold change of expression in the control cells, and were normalized to actin as reference gene. All experiments were performed three times independently, each time in triplicate to confirm the results. Statistical analysis were performed by Student’s *t*-Test. Results were considered significant when * *p* < 0.05, ** *p* < 0.01, *** *p* < 0.001.

The regulation of redox balance was further investigated by evaluating the expression of glutamate-cysteine ligase (GCL), an enzyme involved in glutathione biosynthesis. GCL levels were found significantly overexpressed in B-CPAP and K1 compared to the control cell line (Figure 5A). 

In order to better understand the role of the glutaminolysis pathway in PTC-derived cells, the expression of the enzyme glutaminase-1 (GLS1), which is involved in glutaminolysis, was investigated. Our data demonstrated a significant overexpression of GLS1 in all cancer cell lines compared to control (Figure 5B). Moreover, despite the different genetic background, no significant differences were observed in the GLS1 expression among all PTC-derived cells. 

**Figure 6:**
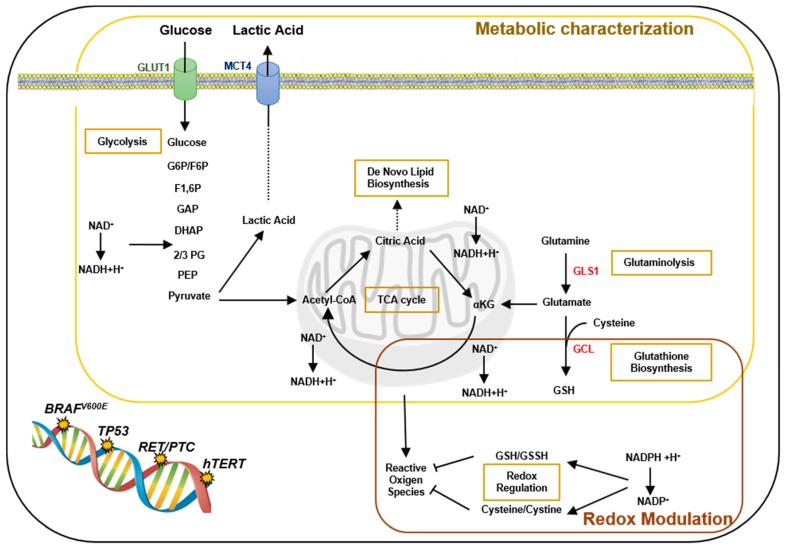
Metabolic pathways, redox regulation and PTC-genetic mutations. Summary of the metabolic alterations and changes in the redox homeostasis described in the studied PTC-derived cells. PTC-derived cells, particularly those cells harboring *BRAF^V600E^* and *TP53*, showed metabolic perturbation in the uptake of nutrients, such as glucose, as further confirmed by the expression of transporters (GLUT1 and MCT4), and alteration in the glycolytic and TCA cycle. TCA cycle alterations are linked to other metabolic and biosynthesis pathways, such as the *de novo* lipid biosynthesis (from citric acid) and glutaminolysis (from αKG, glutamine and glutamate), this was further confirmed by the increased expression of GLS1. Perturbation in the redox modulation has been found in the levels of GSH/GSSG and Cysteine/Cystine ratios, oxidant species and electron carriers (NAD^+^ and NADP^+^). Furthermore, increased expression of GCL enzyme supported the alteration in the redox status of PTC-derived cells.

A summary of all the described metabolic and redox alterations is presented in Figure 6. 

## 3. Discussion 

Metabolic changes and redox perturbation are common features in cancer cells. Both are considered hallmarks of cancer and they are involved in the initiation and progression of the tumor phenotype [1]. Several mutated oncogenes and tumor suppressor genes have been associated to different signaling pathways affecting the tumor cell metabolism and redox status [8]. The aim of this study was to explore and connect the metabolic rewiring and the altered redox balance together with the different PTC-genetic profiles, to better clarify the interplay between oncogenic events, metabolic adaptations and redox perturbation.

### 3.1. Metabolic Alterations in PTC Cells

Our results revealed that metabolites involved in energy production pathways, such as glycolysis, TCA cycle and glutaminolysis, were altered in our PTC-derived cell lines. Particularly, B-CPAP cells, harboring a genetic profile reflecting the aggressiveness of primary tumor from which derived (*BRAF*, *TP53*, and *hTERT* mutations), displayed the most perturbed metabolic phenotype, as demonstrated by altered levels of energetic metabolites (e.g. increase of F6P, DHAP, GAP, 3PG, PEP, and decrease of lactic acid). This higher glycolytic rate in B-CPAP cells suggests a role of the genetic background of these cells in the regulation of metabolic pathways. Indeed, their mutations (*BRAF^V600E^* and *TP53*) were found to be associated with an increased glycolysis, affecting the expression of glycolytic enzymes and glucose transporters [16,17,18], both features required to accomplish the metabolic and energetic demands of proliferating cancer cells [19]. This finding supports the interplay between oncogenic events and metabolic modulation, from which cancer cells take advantages for the growth and survival. These altered metabolic needs reflected also in the perturbations observed for intermediates of the TCA cycle, supporting the hypothesis that cancer cells uncouple glycolysis from the TCA cycle to provide different fuel sources [20,21]. A specific example is citrate, which is an intermediate of several biochemical pathways such as de novo lipogenesis ad sterol biosynthesis [22], and is found to be increased in both K1 and B-CPAP cells. The accumulation of citrate as a promoter of lipid synthesis is confirmed by the concomitant increase of α-KG and glutamine. Indeed, the depletion of citrate from the TCA cycle to sustain lipid biosynthesis is counterbalanced by glutamine with increasing levels of α-KG [23]. The accumulation of fumarate found in B-CPAP cells could be associated with the epithelial-to-mesenchymal transition [24], a phenomenon playing an important role in cancer initiation, growth, invasion, dissemination, and metastasis as well as resistance to therapy [15,25].

An alternative evaluation of the cellular energetic consumption was performed by measuring the glucose uptake and transport. Due to the higher energetic demand, cancer cells usually display an increased glucose uptake facilitated by overexpression of glucose transporters, particularly GLUT1, in the plasma membrane [26]. The accumulation of lactic acid resulting from the increased aerobic glycolytic rate is counterbalanced by an increased expression of monocarboxylate transporters (MCTs), in particular MCT4 [27,28,29]. In order to further confirm the high glycolytic rate, found in PTC-derived cells, and in order to give an insight of nutrient fluxes across membranes, we analyzed, by immunofluorescence, the expression of GLUT1 and MCT4 transporters. Accordingly, B-CPAP cells presented a marked increase in the expression of both GLUT1 and MCT4 in their plasma membrane, as well as the highest glucose uptake rate compared to all other cell lines analyzed, which is in line with their hyper-glycolytic and aggressive phenotype. Moreover, B-CPAP cells showed an increase of glutamine and glutamate, metabolites involved in glutaminolysis. Altogether, these findings support the fundamental role of glutaminolysis in cancer cells and its connection with glycolysis. Indeed, glutaminolysis can be exploited to replenish TCA cycle intermediates, which, as mentioned above, are often reduced by the uncoupling of TCA cycle and glycolysis in tumors [30]. This was further confirmed by the overexpression of GLS1, an enzyme converting glutamine into glutamate, hence providing fuel for the TCA cycle [31], in all PTC-cells compared to control. Interestingly, wild-type p53 plays an important role in glutamine metabolism by activating the glutaminase-2 isoform (GLS2), which promotes energy and antioxidant production [32]. GLS2 resulted to be mainly involved in the redox regulation, promoting glutamate production, which is then directed to the GSH biosynthesis [32]. The expression of GLS1, rather than GLS2, in our case seems to be closely related to the activity of mutant p53 [33], and considering our goal being on the highlighting the connection of mutated *TP53* and glutamolysis, we only focused on the GLS1 expression. The increased expression of GLS1 in PTC-derived cells, particularly in B-CPAP cells, further reflect the interplay among cancer cell metabolism and oncogenic mutations. Particularly, PTC genetic profiles with *BRAF^V600E^* and *TP53* mutations showed the most perturbed metabolic phenotype, confirming the role of these oncogenic events in the reprogramming of cancer metabolism [8].

### 3.2. Redox Alterations in PTC Cells 

In cancer cells, redox regulation covers a fundamental role and results from oncogenic mutations that promote altered metabolism, resulting in increased rates of ROS production [8]. Increasing evidence reported that alteration in the metabolism resulted in a tight control of ROS and antioxidant production [8]. Given the known cross talk and connections between metabolic pathways and redox balance in tumors [8], we decided to explore the redox homeostasis of PTC-derived cells. Significant changes in the redox status were observed, such as decreased levels of both GSH/GSSG and cysteine/cystine ratios as well as significant increase of ROS levels in all PTC-derived cells, in agreement with their perturbed redox condition [34]. Moreover, levels of GSH were found to be lower in PTC-derived cells compared the control cells (data not shown), supporting the presence of oxidative stress. A further symptom of the altered redox balance in PTC is the augmented level in K1 and B-CPAP cells of both NAD^+^ and NADP^+^, key molecules involved in the redox balance and in the antioxidant biosynthesis, respectively [35]. Indeed, NADP^+^ (and its reduced form NADPH) provides reducing power in metabolic reactions and participates to regenerate antioxidant defenses [8]. Furthermore, the same cell lines accumulating NAD^+^ and NADP^+^ presented overexpression of GCL, enzyme responsible for the glutathione biosynthesis and usually overexpressed in cancer [36], confirming the redox perturbation of these tumor cells. The regulation of redox homeostasis is deeply connected to metabolic pathways [8]. Particularly, metabolic reactions such as the conversion of glucose into G6P, isocitric acid into αKG, malic acid into pyruvic acid and glutamine into glutamate, are involved in the regeneration of NADPH to sustain antioxidant defenses [8]. In our data, levels of αKG, glutamine and glutamate were found to be increased in PTC-derived cells, particularly in B-CPAP cells, supporting the involvement of these metabolites in the redox modulation and the interplay between metabolism and redox homeostasis. 

## 4. Conclusions

Our study combines different analytical techniques to gain new insights into the metabolic complexity of papillary thyroid carcinoma and allowed us to confirm the pivotal role of the interplay between the genetic background of tumors and cancer phenotypic features, such as metabolism and redox homeostasis. In conclusion, although the importance of metabolism in cancer cells has been previously pointed out by a number of studies as a promising tool to understand tumor biology, deciphering the metabolic alterations and dependencies of cancer cells still remains a great challenge in this field. On the base of our finding, a multidisciplinary approach in cancer research could be fundamental to define cancer susceptibility and possible dependencies to be targeted. Considering the limit of our study, using a PTC in vitro model with only three PTC-derived cell lines, further experiments are needed, in both cell lines and human tissues, to confirm and to deeply investigate metabolic behavior of PTC.

## 5. Material and Methods 

### 5.1. Cell Culture

The human PTC-derived TPC-1 and B-CPAP cell lines were kindly provided by Dr. Fusco (Medical School, University Federico II of Naples, Naples, Italy), while both PTC-derived K1 cell line and Nthy-ori3–1, Simian Virus 40 (SV40)-immortalized normal human thyrocytes were purchased from the Health Protection Agency Culture Collections [37]. All cell lines were grown as monolayers in culture in Dulbecco’s Modified Eagle’s Medium/Ham’s F-12 (DMEM/F12) supplemented with 10% fetal bovine serum (FBS, Life Technologies, Milan, Italy), 100 UI/mL penicillin and 100 μg/mL streptomycin (Merck, Milan, Italy), at 37 °C in a humidified 5% CO_2_ atmosphere.

### 5.2. Aqueous Metabolites Extraction

Cells were seeded in Petri dishes (100 mm) at the density of 1.8 × 10^6^ cells and let them grow for 48 h. Then cells were harvested by scraping after removal from the dished of the growth medium. Cells were extracted as described previously [38]. Briefly, a mixture of cold methanol and water (80:20 *v*/*v*) was added to the dishes, cells were detached and collected in Eppendorf^TM^ tubes. In order to ensure complete cellular lysis, the extraction was combined with 10 min of ultrasonic treatment at 4 °C. Cell suspensions were centrifuged at 4500 rpm for 30 min at 4 °C. The upper aqueous phase was separated, aliquoted in Eppendorf^TM^ tubes and dried in an Eppendorf^TM^ Concentrator Plus overnight. 

### 5.3. Ultra High Performance Liquid Chromatography-Tandem Mass Spectrometry

Metabolites, in the aqueous dried fraction of the samples, were measured using an ultra-high performance liquid chromatography coupled with a TSQ Quantiva™ Triple Quadrupole Mass Spectrometer (UHPLC-MS/MS) using a targeted approach, in both positive and negative ion mode, with an ESI source. Aqueous metabolites were acquired through selected reaction monitoring (SRM) mass spectrometry analysis, following infusion of standard compounds, using two targeted analysis based on the different polarity of detected compounds. For the first targeted analysis, samples were reconstituted in 200 µL acetonitrile:water (7:3 *v*/*v*) with ammonium carbonate 0.1 M, and then subjected to LC separation on a BEH amide HILIC column (100 × 2.1 mm, 1.7 µm; Waters Ltd) with a flow rate of 600 µL/min and mobile phase consisting of 0.1% of ammonium carbonate water solution and acetonitrile solution. For the second targeted analysis, samples were reconstituted in 0.1% of formic acid in water, and injected using a reversed-phase ACE Excel C18-pfp column (150 × 2.1 mm, 2 µm; ACE). UHPLC was operated at a flow rate of 500 µL/min, with the mobile phase consisting of a solution of 0.1% of formic acid in water (A) and a solution of 0.1% of formic acid in acetonitrile (B) and programmed as follows: starting with 20% of A for 1.50 min, linear increasing from 20% to 60% of A in 2.5 min, for one minute kept at this percentage, and brought back to initial condition after 0.1 min. Both mass spectrometry analyses were performed using a standard-mix containing 10 uM of each of the following isotope-labelled compound: [^13^C, ^15^N] L-proline, L-leucine-d10, L-Valine-d8, L-phenylalanine d5, Succinic acid-^13^C and Serotonine-d4 (Merck, Gillingham, Dorset, UK) and [^13^C, ^15^N] L-glutamate (Cambridge Isotope Laboratories, Andover, MA, USA). The Xcalibur software (Thermos Fisher Scientific, Waltham, MA, USA) was used for data acquisition. Putative recognition of all detected metabolites was performed by inspection of the spectra deriving from a targeted MS/MS analysis. Metabolites with different isoforms (2/3-phosphoglicerate and glucose-6-phosphate/fructose-6-phosphate) could not be chromatographically resolved, thus peaks of these metabolites were considered as the sum of both isoforms. Retention times, calculated masses, mass fragments and acquisition ion mode of the compounds are reported in Table 2. Peak areas for each detected metabolite were normalized by total area and reported as ranks in the bar graph.

**Immunofluorescence**. Cells were enzymatically dissociated, cultured on previously sterilized slides, and placed in square tissue culture dishes (quadriPERM^®^, Sarstedt AG & Co, Nümbrecht, Germany). Cell cultures were maintained for 16 h at 37 °C in a humidified 5% CO_2_ atmosphere, followed by fixation with 4% paraformaldehyde for 10 min at room temperature. Immunostaining was performed as previously described [15] using rabbit polyclonal anti-GLUT1 (1:200; Abcam, CA, USA) and rabbit polyclonal anti MCT4 (1:100; Abcam, Cambridge, UK) antibodies. Alexa-conjugated (Alexa Fluor 488 or 594, Life Technologies) goat anti-rabbit IgG was 0used as secondary antibodies. Nuclei were counterstained with 4′,6-diamidino-2-phenylindole (DAPI).

**Glucose uptake assay**. Cells were seeded in 96-well plates (1 × 10^5^ cells/mL) and incubated for 24 h. After incubation, plates were washed with PBS, followed by the addition of 50 µM of 2-[N-(7-nitrobenz-2-oxa-1, 3-diazol-4-yl) amino]-2-deoxy-Dglucose (2-NBDG, fluorescently-tagged glucose derivative, N13195; Invitrogen), which was incubated for 30 min. External 2-NBDG was washed off and replaced with PBS. Relative glucose uptake was measured by reading the fluorescence emitted using a micro plate reader (Infinite 200, Tecan, Salzburg, Austria) at a controlled temperature of 37 °C. The measurement was performed using an excitation of 485 nm and an emission of 530 nm.

**Protein Quantification.** Cells were seeded in 6-well plates (1 × 10^5^ cells/mL) and incubated for 48 h and 72 h for western blot and intracellular aminothyiols determination, respectively. Then cells were washed twice with ice-cold PBS and lysed with CelLytic M (Merck, Milan, Italy) supplemented with protease and phosphatase inhibitor mini tables (Thermos Fisher Scientific, Waltham, MA, USA). Cell were then incubated for 15 min on ice before centrifugation at 12,500 *g* at 4 °C for 7 min and stored at –20 °C prior quantification. Protein quantification was performed by using Bradford Assay. Calibration curve was generated using bovine serum albumin (BSA) in lysis buffer, at different concentrations (0.1 mg/mL–10 mg/mL). 500 μL of Bradford reagent solution was incubated with either 10 μL of standard or protein sample at room temperature for 5 min. The absorbance was measured at 595 nm using a micro plate reader (Infinite 200, Tecan, Salzburg, Austria) at a controlled temperature of 37 °C. The equation of the line of the standard curve was used to calculate the protein concentration of the cell samples.

**Determination of intracellular aminothyols level.** GSH, GSSG, Cysteine and Cystine levels were determined in B-CPAP, K1, TPC-1 and Nthy-ori 3-1 cell lines with EC-HPLC quantification, using the method described by Khan et al [39]. Cells were seeded in 6-well plates (1 × 10^5^ cells/mL) and incubated for 72 h. After incubation, cells were scraped and extracted with 150 μL of 10% meta-phosphoric acid and 150 μL of 0.05% trifluoroacetic acid (TFA) (Merck, Milan, Italy) solution. The solution was centrifuged and the resulting clear supernatant was injected into the Agilent 1260 infinity HPLC system (Agilent Technologies, Santa Clara, USA) equipped with an electrochemical detector (DECADE II Antec, Leyden, The Netherlands) and an Agilent interface 35900E. A calibration curve was created using standards of GSH, GSSG, Cysteine and Cystine (Merck, Milan, Italy) injected at different concentrations. Data were collected and expressed as ng of GSH, GSSG, Cysteine and Cystine to μg of protein(s) measured by Bradford assay as described above.

**Determination of intracellular ROS production.** Intracellular ROS were measured in B-CPAP, K1, TPC-1 and immortalized non tumoral Nthy-ori 3-1 cell lines, seeded in 96-well plates (7.5 × 10^3^ cells/mL), washed with PBS solution and incubated for 30 min with 2′,7′-dichlorofluorescin diacetate (H_2_-DCF-DA, Merck, Milan, Italy) 10 μM in PBS, as previously described [40]. H_2_-DCF-DA was then removed and cells were exposed to PBS. ROS production was determined by measuring the fluorescence emitted (excitation of 490 nm and an emission of 520 nm) after 2 h using a micro plate reader (Infinite 200, Tecan, Salzburg, Austria) at a controlled temperature of 37 °C. 

**Western blot analysis.** Immunoblotting experiments were performed with the following antibodies: mouse monoclonal antibody directed against actin (clone AC-40) (Merck, Milan, Italy) and two rabbit polyclonal antibodies directed against Glutaminase (ab93434; Abcam, Cambridge, UK) and GCL (Merck, Milan, Italy), respectively. 

For expression analysis, equal amounts (20 µg/lane) of proteins were electrophoresed on SDS-10% polyacrylamide gels (Bio-Rad, Hercules, CA, USA). Protein-sample preparation was used for all gels, which were electrotransferred onto nitrocellulose membrane (Osmonics, Westborough, MA, USA). Membranes were stained with 0.5% (wt/vol.) Ponceau S red (ICN Biomedicals, Aurora, Ohio, USA), blocked in TBS containing 0.05% Tween 20 (Merck, Saint Louis, MO, USA) and 5% BSA (Sigma Aldrich), and incubated with the specific primary antibodies diluted in blocking buffer. Membranes were incubated with anti-mouse or anti-rabbit horseradish peroxidase conjugated IgGs (Santa Cruz Biotechnology, CA, USA). Immunoreactive bands were identified with a chemiluminescence detection system, as described by the manufacturer (Supersignal Substrate, Pierce, Rockford, IL, USA). Densitometric analysis of immunoreactive bands was performed using the program ImageJ (JAVA, Wayne Rasband, National Institute of Health, USA).

**Statistical Analysis.** Experimental results were subjected to Student t-Test with the GraphPad prism v5.0 software (GraphPad software, La Jolla, USA). Statistical significance was calculated comparing average of results from each PTC-derived cells with control cells. Data are presented as means ± standard deviation. 

## Figures and Tables

**Table 1:** Mutational status of cell lines.

*Cell Lines*	*Tissue Origin*	*hTERT*	*BRAF^V600E^*	*RET*/*PTC*	*TP53*
B-CPAP	PTC	C228T	GTG -> GAG *	Wt	GAC -> TAC
K1	PTC	C228T	GTG -> GAG **	Wt	wt
TPC-1	PTC	C228T	wt	*RET*/*PTC1*	wt
Nthy-ori 3-1	NTT	wt	wt	wt	wt

* homozygous; ** heterozygous; NTT: normal thyroid tissue; PTC: papillary thyroid carcinoma; wt: wild type [15].

**Table 2:** Mass spectral data of detected compounds using UHPLC/MS-MS.

Compound	RT	Precursorion (m/z)	Production (m/z)	Polarity	Column
**2/3 Phosphoglycerate**	4.02	185.000	97.065	Negative	BEH amide
**Acetyl-CoA**	3.65	810.225	303.049	Positive	BEH amide
**α-ketoglutarate**	3.35	145	101.123	Negative	BEH amide
**Citric acid**	1.32	191	111	Negative	C18-pfp
**Dihydroxyacetone phosphate**	3.88	169	97.143	Negative	BEH amide
**Glucose-6-phosphate/Fructose 6 phosphate**	4.05	259	97.077	Negative	BEH amide
**Fructose bisphosphate**	4.24	339	97.084	Negative	BEH amide
**Fumaric Acid**	3.48	115	71.172	Negative	BEH amide
**Glucose**	2	179	89	Positive	BEH amide
**Glutamic Acid**	0.74	148	84.15	Positive	C18-pfp
**Glutamine**	0.72	153.1	89.169	Positive	C18-pfp
**Glyceraldhehide-3-phosphate**	3.83	169.009	97	Negative	BEH amide
**Isocitric acid**	1.71	191	111	Negative	C18-pfp
**Lactic Acid**	2.5	89	43	Negative	BEH amide
**Malic Acid**	3.67	133	115.081	Negative	BEH amide
**NAD^+^**	3.82	664.1	136.237	Positive	BEH amide
**NADP^+^**	4.15	744.1	136.2	Positive	BEH amide
**Oxaloacetate**	3.34	131	87.084	Negative	BEH amide
**Phosphoenolpyruvate**	3.94	167	79.081	Negative	BEH amide
**Pyruvic acid**	2.75	87	43	Negative	BEH amide
**Succinic acid**	3.51	117	73	Negative	BEH amide
**Succynil-CoA**	3.92	868.1	361.214	Positive	BEH amide

Compound name, retention time, calculated masses, mass fragments, polarity and column for all detected metabolites.

## Supplementary Materials

The following are available online at http://www.mdpi.com/2218-1989/9/2/23/s1, Figure S1: Nucleotides levels in PTC-derived cells. Figure S2: Amino acids levels in PTC-derived cells. Table S1: Holm-Bonferroni sequential correction of *p* values obtained from unpaired Student *t*-test.
