# Supplementary material for: Crosstalk between Metabolic Alterations and Altered Redox Balance in PTC-Derived Cell Lines
Source: Metabolites. 2019 Feb 1;9(2):23. doi: 10.3390/metabo9020023 (PMC6409540; doi:10.3390/metabo9020023)
Supplement: Supplementary file 1 [file metabolites-09-00023-s001.zip › supplementary/metabolites-421682-supplementary.pdf]

Supplementary Files

Table 1. Holm-Bonferroni sequential correction of p values obtained from unpaired Student T-test.

Compound	TPC-1 vs. Nthy-ori3-1	K1 vs. Nthy-ori3-1	B-CPAP vs. Nthy-ori3-1
Glucose	0.0579	NS	NS
F6P	0.0064	NS	0.0015
F1,6P	0.0294	NS	NS
GAP	0.0294	0.0024	0.0015
DHAP	NS	NS	NS
2-PG/3-PG	0.0018	0.0015	0.0144
PEP	0.0011	0.0015	0.0015
Pyruvic acid	0.0224	0.011	NS
Lactic acid	NS	NS	NS
Acetyl-CoA	0.0579	NS	0.0196
Citrate	NS	NS	0.013
Isocitrate	NS	NS	0.016
a-ketoglutaric acid	0.0294	0.0304	0.0033
Succinyl CoA	0.0579	NS	NS
Succinic acid	NS	0.0261	NS
Fumaric acid	NS	0.012	0.0425
Malic acid	NS	NS	NS
Oxoloacetic acid	0.0579	NS	0.0324
Glutamine	0.0011	0.0015	0.0024
Glutamate	NS	0.0304	NS
