# Supplementary figures and images for: Crosstalk between Metabolic Alterations and Altered Redox Balance in PTC-Derived Cell Lines
Source: Metabolites. 2019 Feb 1;9(2):23. doi: 10.3390/metabo9020023 (PMC6409540; doi:10.3390/metabo9020023)

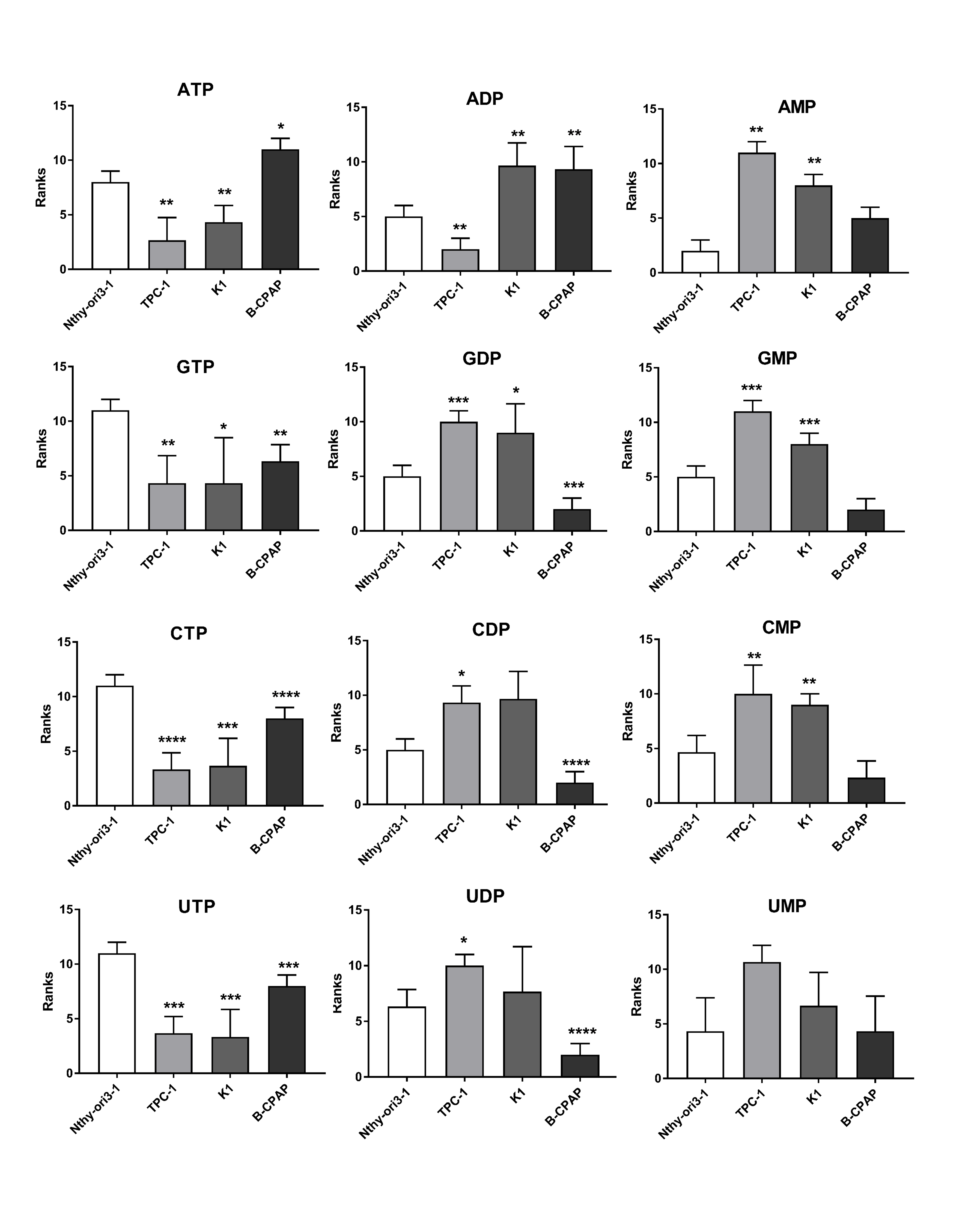

Supplement: Supplementary file 1 [file metabolites-09-00023-s001.zip › supplementary/Figure S1.tif]

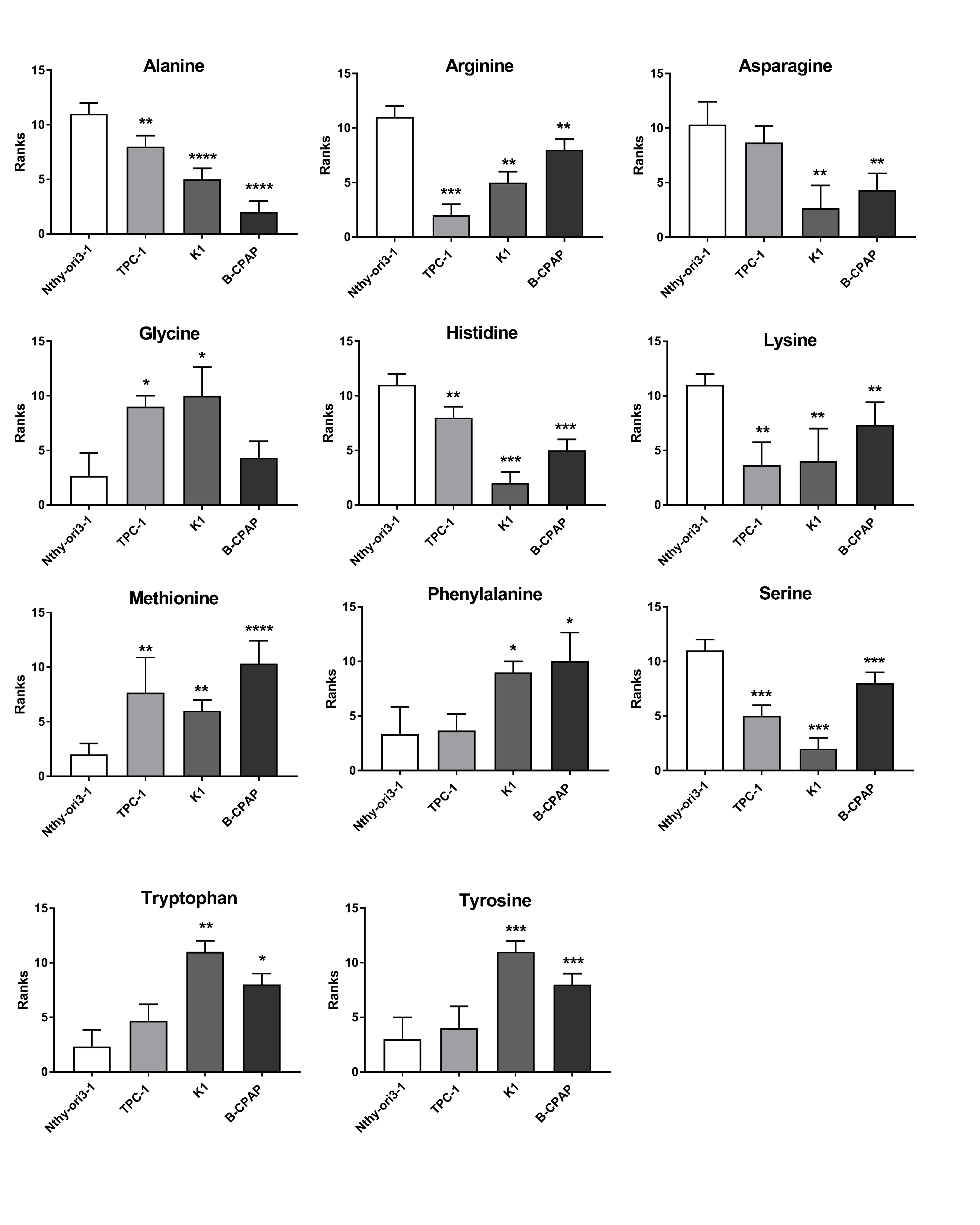

Supplement: Supplementary file 1 [file metabolites-09-00023-s001.zip › supplementary/Figure S2.tif]
